# Cost-effectiveness Analysis of Radium-223 Dichloride in Metastatic Castration-Resistant Prostate Cancer Patients Without Previous Chemotherapy Treatment in Spain

**DOI:** 10.36469/9777

**Published:** 2018-01-29

**Authors:** Eva Tirado Mercier, Daniel Callejo Velasco, Marta Rubio Cabezas, Cristina Moretones Agut, Meritxell Granell Villalón

**Affiliations:** 1 Pharmaceuticals Division Bayer Hispania, SL, Barcelona, Spain; 2 IQVIA, Madrid, Spain

**Keywords:** metastatic castration-resistant prostate cancer, radium-223, cost-effectiveness analysis

## Abstract

**Purpose:**

To perform a cost-effectiveness analysis of radium-223 plus Best Supportive Care (BSC) compared to BSC in the treatment of patients with metastatic castration-resistant prostate cancer (mCRPC) and without previous docetaxel treatment in Spain.

**Design and methods:**

A Markov model was developed to compare radium-223 versus BSC and to accrue the health outcomes and costs of a simulated cohort of mCRPC patients. Quality-adjusted life year (QALY) and life year (LY) were selected as health outcomes to measure the effectiveness of treatment alternatives. Main health resource use and efficacy inputs were obtained from a randomized controlled trial comparing radium-223 versus placebo. Unit costs were retrieved from Spanish databases and published sources. One-way and probabilistic sensitivity analyses were carried out to assess uncertainty.

**Results:**

Total costs and QALYs were €65 067 and 1.12 QALYs for radium-223 and €55 437 and 0.77 QALYs for BSC. Therefore, incremental costs per QALY were €27 606. The sensitivity analysis showed that with a willingness-to-pay threshold of €30 000 per QALY, radium-223 would have a probability of 48% of being cost-effective compared to BSC.

**Conclusions:**

Although results must be assessed with caution, from the Spanish National Health System perspective and based on the results of the present analysis, radium-223 could be a suitable option of health resources’ utilization for end of life mCRPC without previous docetaxel treatment, subject to a moderate level of uncertainty.

## Introduction

Prostate cancer is the second most common cancer among men, after skin cancer1 and the fifth-leading cause of death, accounting for 6.6% of all cancer deaths, worldwide. Mortality is higher in developing countries, doing special mention to the black populations.^2^ Conversely, the lowest mortality rate belongs to the Asian population.^1^

In Spain, prostate cancer is the most frequently diagnosed cancer in men and the second one when both sexes are considered. In 2010 there was an incidence of 27 853 new cases of prostate cancer, representing 12.9% of all diagnosed tumors. In men, prostate cancer is the third leading cause of death, which corresponds to 8.6% of the total deaths caused by cancer.^3^

Age, family history and race are the only three risk factors which have shown to be related to the likelihood of developing prostate cancer.^4^

The management of prostate cancer has been based on local treatments (radical prostatectomy and radiotherapy) and androgen deprivation that managed to control the disease temporarily. However, over time, patients begin to show signs of disease progression, with increased Prostate Specific Antigen. This state is known as castration-resistant prostate cancer (CRPC), where the prognosis is worse than first stage prostate cancer, and the median overall survival (OS) ranges between 9 to 30 months.^5^ Although at the time of diagnosis, between 3% and 8% of patients have bone symptoms, of those, up to 90% will develop bone metastasis in the next 15 years (mCRPC).^5^ In this stage of the disease appears a set of complications known as a symptomatic skeletal event (SSE). SSE includes pathologic fractures, spinal cord compression, bone surgery and radiotherapy in the bones.^6^ On average, a patient with mCRPC will have a SSE every 3–6 months (IPT radium-223 dichloride, 2015). SSEs are associated with impaired mobility, reduced quality of life and increased health care costs.^7^

Currently, there are effective treatments to reduce pain and delay the SSE, such as denosumab and zoledronic acid, but these treatments do not offer a benefit on patient survival.^7^ Conversely, available active treatments have shown survival benefits in mCRPC without proving benefit on SSE. These active treatments include chemotherapy treatments as docetaxel^8^ and cabazitaxel^9^, and hormone therapies as abiraterone acetate (hereinafter, abiraterone)^10^ and enzalutamide.^11^ Recently radium-223 dichloride (hereinafter, radium-223), a bone-targeted alpha therapy, has shown impact on OS and delay of disease progression and SSEs.^6^

Therefore, during the last few years, the therapeutic options for the treatment of mCRPC has been expanded. Despite this fact, we cannot forget that health systems have limited resources, and therefore, the efficiency and cost-effectiveness of the new treatments have become a requirement in most of the countries.

Therefore, the aim of this paper is to assess the cost-effectiveness of radium-223 compared with BSC in a population of patients with mCRPC who were previously treated with docetaxel in Spain.

## Design and Methods

To meet the objectives previously described, a cost-effectiveness analysis has been carried out estimating the costs and the health outcomes achieved by patients treated with the new intervention, radium-223 plus BSC versus BSC alone.

The population included in the assessment was those mCRPC patients without previous treatment with docetaxel, neither other chemotherapy. A health system perspective was adopted to conduct the economic evaluation.

**Figure 1:**
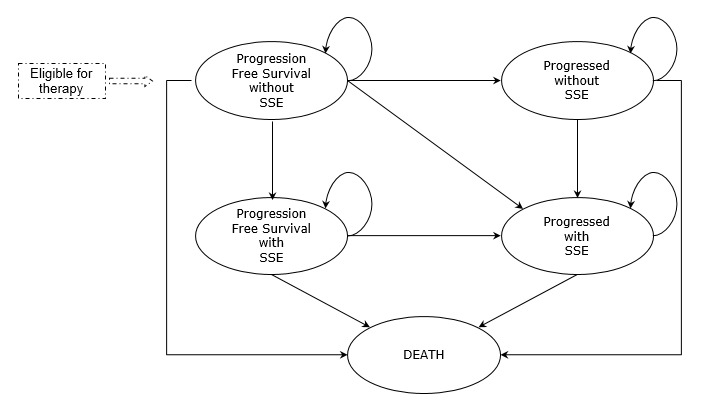
Markov Model for mCRPC SSE: Symptomatic Skeletal Event

A pharmacoeconomic Markov model has been developed in Microsoft Excel® to ensure transparency and flexibility. The model includes five mutually exclusive health states that allows simulate the evolution of mCRPC (Figure 1). All the patients initiate the simulation in the health state called “progression-free survival without SSE”. At the end of each cycle patients can move to one of the other four health states: “progression without SSE”, “progression-free survival with SSE”, “progression with SSE” and “death” or remain in their initial health state. The model used a cycle length of 1 week to allow an adequate simulation of the rapid disease progression.

The time horizon for the assessment was 10 years. It was deemed enough due to the fact that live expectancy in this population is limited and therefore 10 years are considered a lifetime horizon allowing to capture all costs and health benefits of both comparators.

### Inputs Used to Populate the Model

#### Effectiveness

The effectiveness of the alternative treatments has been based on one randomized clinical trial, using the information on the target pre-chemotherapy population.^6^

The ALSYMPCA trial is a phase III randomized, double-blind, multi-country trial to assess the efficacy and safety of radium-223 plus BSC for the treatment of mCRPC patients versus placebo plus BSC.

In brief, in the ALSYMPCA trial a total of 921 patients were recruited, of those, 526 received previous treatment with docetaxel, while the 395 remaining patients had not received previous treatment with chemotherapy (no previous docetaxel group). There were no significant differences between these two groups regarding basal characteristics of patients beyond the previous use of docetaxel. The patients were randomized in a ratio 2:1, to radium-223 (55 KBq/Kg every 28 days for a maximum of 6 cycles) or placebo, respectively. For the present evaluation, only the data of the no previous docetaxel subgroup of patients has been used.

The BSC was administered to both treatment arms and defined as the routine treatment provided at each clinical centre, including: external radiotherapy, corticosteroids, antiandrogens, ketoconazole, diethyl estilbestrol, or estramusina.^6^

The primary and the secondary objectives of the ALSYMPCA trial were achieved: an OS with reduced risk of death by 30% and time to first SSE, time to increase alkaline phosphatase, time to prostate-specific antigen increase, quality of life, and safety were favorable to patients randomized to radium-223. Additional information can be seen elsewhere.^6^

The main variables that determine treatment efficacy, time to death, time to disease progression and time to SSE were assessed through survival analyses. To extrapolate the treatment efficacy observed during the study follow-up to 10 years of the time horizon used in the model, parametric survival curves were used. The estimation was carried out from the individual data of the subgroup of patients without previous docetaxel treatment, included in the ALSYMPCA study. Several survival models were tested: exponential, log-normal, log-logistic and Weibull. The log-normal function had been selected for the economic evaluation for being the one that best fits to the trial data, based on visual and statistical criteria, Akaike Information Criterion (AIC). The clinical inputs used to populate the model are shown in Table 1.

#### Utility Scores/Quality of Life

In cost-utility analysis QALYs are calculated as a standard method to quantify the quality of life in each health state. QALYs are calculated by multiplying the utility value of the health state (a measure of quality of life reported by the patient in a questionnaire referred to his health status) for the time spent in each health state. The quality of life measures were also obtained from ALSYMPCA trial. Disease progression and SSE have an impact on quality of life.

In the ALSYMPCA trial, quality of life of patients were obtained with the European Quality of Life-5 Dimensions (EQ-5D) questionnaire collected at different time points during the trial: in the basal visit, in two visits along the treatment period (weeks 16 and 24) and then during the follow-up visits. Utility observations that were missing or where utility date was missing were excluded. By the end of the study, the average number of utility score measurements was 3.8 for radium-223 patients and 3.2 for placebo patients (3178 utility scores over 882 patients). During the treatment period the utility values for patients treated with radium-223 and BSC were different, but once the disease advance it was assumed the same utility values for both treatments. The mean observed values by treatment and progression state are shown in Table 2.

#### Resource Use and Costs

The use of health resources has also been collected in the ALSYMPCA trial. It includes hospital days, day care use and physician visits. Other health resources, distinguishing between pre-progression and post-progression health states but not according to the treatment, are valued as described by expert opinion.

Unit costs were retrieved from Spanish databases and published sources.^12^,^13^

The drug prices were valued at ex-factory price notified applying the 7.5% deduction of established by Royal Decree Law 8/2010 of 20 May when necessary.

Other unit costs included in the model were: the costs of administration (in the case of intravenous infusion (IV) of radium-223); cost of drugs to treat adverse events; cost of procedures for monitoring patients; cost of the treatments of SSE; cost of the “end of life” treatments; and costs of other resources such as the day of hospitalization, day-care unit and physician visits. All data referring to resource use and unit costs are collected in Table 3.

#### Analysis

The base case analysis was conducted from a National Health System perspective and used QALYs as health outcome measure, and the following results for each comparator are displayed, total discounted cost, total discounted QALYs, total life years, incremental costs, incremental QALYs and incremental life-years. Finally, incremental cost-effectiveness ratio (ICER) was estimated as follows:

$$\text{ICER}=\frac{{\text{cost}}_{\text{Radium}-223}-{\text{cost}}_{\text{BSC}}}{{\text{QALY}}_{\text{Radium}-223}-{\text{QALY}}_{\text{BSC}}}$$

Additionally, cost breakdown was also presented.

#### Uncertainty

Because of the parameters uncertainty inherent in any model, it is recommended to perform a sensitivity analysis to test the robustness of the results, that is, to what extent the variation of the main variables can alter the final results. One-way and probabilistic sensitivity analyses were conducted to assess the uncertainty associated with parameter estimations and the robustness of the base case results.

A tornado diagram presenting the 10 parameters which have a greater impact in the results of the economic evaluation was developed.

The probabilistic sensitivity analysis was performed by means of 1000 cohort simulations, and results were displayed by a cost-effectiveness acceptability curve. Gamma and beta distributions were used to run simulations; gamma distribution for resource use and unit costs; and beta distribution for rates, probabilities and utility scores.

Future costs and health outcomes were discounted at an annual rate of 3% in the base case analysis. In one-way sensitivity analysis, rates of 0% and 5% were applied as recommended in Spanish guidelines.^14^

## Results

### Base Case

Patients treated with radium-223 achieve a mean of 1.12 QALYs, improving in 0.35 the results achieved by patients treated with BSC. The total cost per patient treated with radium-223 was €65 067; this involves an increment of €9631 cost for a patient treated with BSC. Thus, the ICER of radium-223 versus BSC for the treatment of mCRPC without previous chemotherapy treatment was €27 606 per QALY gained (Table 4).

The incremental benefit in LYs gained achieved by radium-223 was 0.40.

A detailed analysis of cost breakdown (Table 5), shows that radium-223 increased the drug costs in €27 195 but allows to save €14 643 in the management of the patient, plus €3255 in avoided hospitalizations. Other cost groups were similar between treatments.

### One-way Sensitivity Analysis

The survival curves used to model progression and OS show a great impact on the results (Table 6).

**Figure 2:**
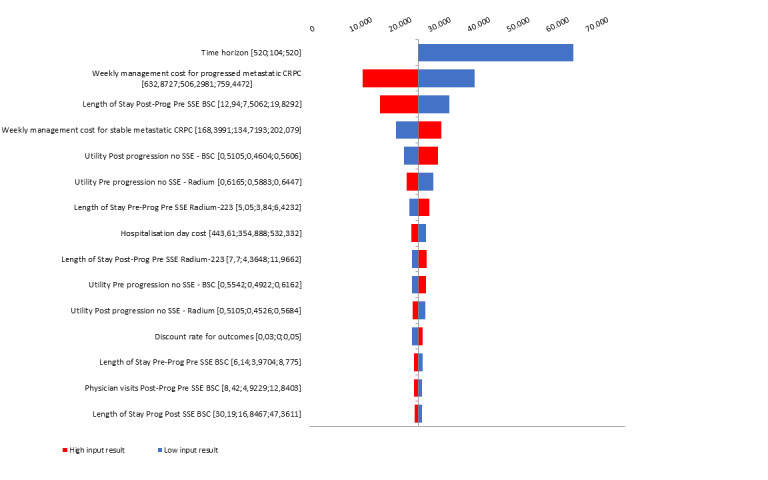
One-way Sensitivity Analysis, Tornado Diagram BSC: best supportive care; CRPC: castration-resistant prostate cancer; SSE: symptomatic skeletal event

The one-way sensitivity analysis (OWSA) showed that time horizon was the most influential parameter, the ICER of radium-223 raised with the decrease of the time horizon. It seems evident because in shorter time horizons it is not allowed to retrieve the whole clinical benefit, but drug costs were generated. Other parameters with a remarkable impact on results were weekly management cost and length of stay in postprogressed patients; the smaller they were the less efficient the radium-223 was, since less value has the delay in progression achieved with radium-223 (Figure 2).

### Probabilistic Sensitivity Analysis

**Figure 3:**
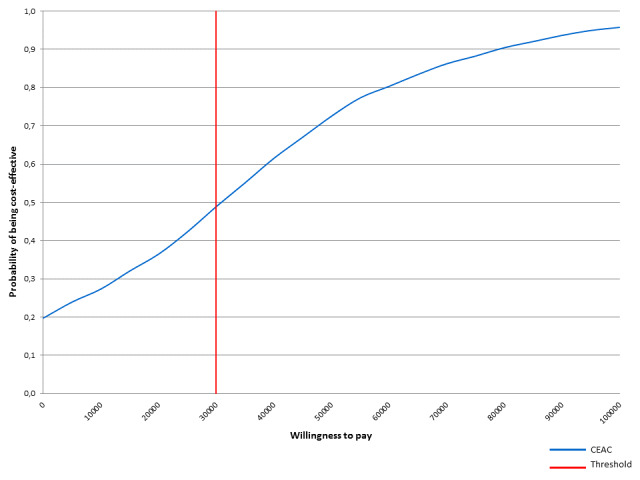
Cost-effectiveness Acceptability Curve Note: The cost-effectiveness acceptability curve (CEAC) is a graph summarizing the impact of uncertainty on the result of an economic evaluation. The graph plots a range of cost-effectiveness thresholds on the horizontal axis against the probability that the intervention will be cost-effective at that threshold on the vertical axis. It summarizes the results of the probabilistic sensitivity analysis. The CEAC would help the decision-maker to understand the uncertainty associated with making a particular decision to reimburse or reject a new drug.

In the cost-effectiveness acceptability curve (CEAC; Figure 3) we can see that the probability of being costeffective for radium-223 was 48% with a willingness-to-pay of €30 000 per QALY, suggesting there was a high uncertainty regarding which alternative compared was the best option for mCRPC patients.

## Discussion

Our National Health System is a system of limited resources, and each year must attend a multitude of applications for approval of new health interventions. In the pursuit of sustainability of the system, the government must assess what additional costs are associated with new alternatives and if the benefits derived justify such investments. This leads to a growing need for economic evaluations. That is why the growing need for economic evaluations that facilitate decision-making regarding new drugs and medical devices.

In this analysis of cost-effectiveness analysis, we evaluated radium-233 versus BSC among patients with mCRPC without previous use of docetaxel has been evaluated. Our analysis, which has been conducted from a health system perspective, indicates that the ICER is below €30 000 per QALY gained.

No previous economic evaluations of radium-233 in Spain were found. However, they exist in other settings. In the article written by Norum *et al*., concluded that the use of radium-223 was not cost-effective or at least there were not enough data to conclude that it was.^15^ Renzulli *et al*., stated in his article the experience of a multidisciplinary approach in the treatment of mCRPC, and concludes that radium-223 has demonstrated overall survival and delayed onset of SSE regardless of whether it was administered or not previously to docetaxel.^16^ Gaultney *et al*., developed a Markov model for analysis of cost-effectiveness analysis with radium-223 and several comparators in the Netherlands.^17^ The results conclude that, given abiraterone, radium-223 is cost-effective, that is, radium-223 is more efficient but also more expensive than abiraterone, although below the threshold of willingness-to-pay. Finally, Henricks *et al.*, work also developed a Markov model where the delay in the onset of SSE and the costs associated with hospitalization by radium-223 make it to be cost-effective compared to BSC.^18^

The robustness of our base case results were tested in the sensitivity analysis. In the one-way sensitivity analysis, the time horizon considered was the parameter with a higher impact on the results was the time horizon considered: the shorter it was the less efficient radium-223 resulted. The higher uncertainty is related with the type of survival curve selected to extrapolate trial results on a longer time horizon, but in the base case analysis the Akaike Information Criterion was used to determine which parametric curve better fits the Kaplan-Meier data.

In the probabilistic sensitivity analysis and considering a willingness-to-pay of €30 000 per QALY, there is a probability of 48% that radium-233 is cost-effective against its comparator. This probability increases to 66% and with a willingness to pay of €45 000 per QALY. For Spain, the threshold of efficiency, from which, a new health intervention is considered cost-effective, the literature so far provided us with the limit of efficiency in €30 000 – €45 000 per QALY. Referring to our country, the literature has provided the efficiency threshold of €20 000 – €45 000 per QALY, when establishing a threshold of efficiency from which a new health intervention is considered cost-effective.^19^–^21^

There are a number of limitations to be noted. The first one was the use of same utility scores for both arms once the disease advanced. Utility scores included in the model were collected from patients included in the ALSYMPCA trial.^6^ This allowed the use of different utility scores in both arms during the active treatment period, but not longer. However, after the progression of the disease, patients discontinued their treatments, so the quality of life should be similar regardless of their initial treatments. Another limitation was the fact that the use of healthcare resources was also collected from the ALSYMPCA trial. Therefore, the typical standard of care differs from the care provided during a trial, which may have biased the results. Nevertheless, utility scores are considered a better proxy than to define healthcare resource use by an Advisory Board. The third limitation was the use of BSC instead of another active treatment. Active treatment use involves the development of an indirect meta-analysis comparison. In Spain, abiraterone should be a possible alternative therapy for mCRPC patients without previous docetaxel treatment. However, populations included in radium-223 and abiraterone trials were not considered similar enough to perform indirect comparisons.^6^,^22^

In conclusion, from the Spanish National Health System perspective and based on the results of the present analysis, radium-223 could be a suitable option of health resource utilization for end-of-life mCRPC patients without previous docetaxel treatment, subject to a moderate level of uncertainty.

## Figures and Tables

**Table 1:** Clinical Inputs Used to Populate the Model

	Base Case Value	Lower Value OWSA	Upper Value OWSA
**Demographics**			
Weight	82.90	81.95	83.85
Body Surface Area	1.86	1.84	1.88
Mean number of injections radium-223	6.00	5.87	6.13
**Efficacy data**			
Overall Survival BSC scale	3.8849		
Overall Survival BSC shape	−0.1165		
Overall Survival radium-223 scale	4.1465		
Overall Survival radium-223 shape	−0.1035		
Progression Free Survival BSC scale	2.5385		
Progression Free Survival BSC shape	−0.4314		
Progression Free Survival radium-223 scale	3.8067		
Progression Free Survival radium-223 shape	−0.0116		
Symptomatic skeletal event BSC scale	3.8319		
Symptomatic skeletal event BSC shape	0.2994		
Symptomatic skeletal event radium-223 scale	4.2223		
Symptomatic skeletal event radium-223 shape	0.2494		
**Adverse Event**			
Anaemia BSC	0.58%	0.40%	0.76%
Anaemia radium-223	0.07%	0.03%	0.11%
Bone Pain BSC	1.22%	0.96%	1.51%
Bone Pain radium-223	0.02%	0.00%	0.04%
Diarrhoea BSC	0.07%	0.01%	0.13%
Diarrhoea radium-223	0.97%	0.80%	1.14%
Fatigue BSC	0.26%	0.14%	0.38%
Fatigue radium-223	0.01%	0.00%	0.02%
Febrile neutropenia BSC	0.01%	0.00%	0.04%
Febrile neutropenia radium-223	0.09%	0.04%	0.14%
Hypokalemia BSC	0.01%	0.00%	0.04%
Hypokalemia radium-223	0.27%	0.19%	0.36%
Nausea BSC	0.07%	0.01%	0.13%
Nausea radium-223	0.17%	0.10%	0.24%
Neutropenia BSC	0.03%	0.00%	0.07%
**Neutropenia radium-223**			
Thrombocytopenia BSC	0.08%	0.02%	0.15%
Thrombocytopenia radium-223	0.57%	0.45%	0.70%
Vomiting BSC	0.10%	0.03%	0.17%
Vomiting radium-223	0.07%	0.03%	0.11%
**Distribution of Skeletal Related Events**			
External beam radiation BSC	78.51%		
External beam radiation radium-223	79.23%		
Pathologic bone fracture BSC	9.92%		
Pathologic bone fracture radium-223	12.08%		
Spinal cord compression BSC	11.57%		
Spinal Cord Compression radium-223	7.73%		
Surgical intervention BSC	0.00%		
Surgical intervention radium-223	0.97%		
Second line treatment BSC	53.70%	0.38	0.70
Second line treatment radium-223	62.70%	0.44	0.82

BSC: best supportive care; OWSA: one-way sensitivity analysis

Note: Lower and upper values in one-way sensitivity analysis are 95% confidence interval when available or a range ± 30% over base case value.

**Table 2:** Utilities to Radium-223 and Best Supportive Care

Utility	Base Case	Lower Value OWSA	Upper Value OWSA
Progression-free no SSE BSC	0.554	0.492	0.616
Progression-free no SSE radium-223	0.617	0.588	0.645
Progression no SSE BSC	0.511	0.460	0.561
Progression no SSE radium-223	0.511	0.453	0.568
Progression-free SSE BSC	0.475	0.444	0.506
Progression-free SSE radium-223	0.475	0.444	0.506
Progression SSE BSC	0.474	0.443	0.505
Progression SSE radium-223	0.474	0.443	0.505

BSC: best supportive care; OWSA: one way sensitivity analysis; SSE: symptomatic skeletal event

Note: Range tested in one-way sensitivity analysis was ± 30% over base case value.

**Table 3:** Resource Use and Unit Costs

	Base Case	Lower Value OWSA	Upper Value OWSA
**Resource use**			
**Proportion of patients requiring hospitalization**			
Neutropenia	0.05	0.04	0.06
Febrile neutropenia	0.50	0.40	0.60
Fatigue	0.30	0.24	0.36
Nausea	0.45	0.36	0.54
Vomiting	0.65	0.52	0.78
Anaemia	0.38	0.30	0.46
Thrombocytopenia	0.13	0.10	0.16
Hypokalemia	0.75	0.60	0.90
Bone Pain	0.34	0.27	0.41
Diarrhoea	0.43	0.34	0.52
**Management resource use**			
**Stable disease**			
Physician outpatient visit (per week)	0.08	0.06	0.10
Imaging CT scan – abdominal (per week)	0.04	0.03	0.05
Imaging bone scan (per week)	0.06	0.05	0.07
Complete blood count (per week)	0.08	0.06	0.10
Prostatic Specific Antigen (per week)	0.08	0.06	0.10
**Length of stay (days per year) ALP based**			
Pre-SSE radium-223	5.05	3.84	6.42
Pre-SSE BSC	6.14	3.97	8.78
Post-SSE radium-223	20.78	13.90	29.02
Post-SSE BSC	41.18	26.24	59.43
**Care Center Use (days per year) ALP based**			
Pre-SSE radium-223	0.43	0.16	0.82
Pre-SSE BSC	0.56	0.19	1.12
Post-SSE radium-223	1.38	0.38	3.02
Post-SSE BSC	1.05	0.09	3.19
**Physician visits (visits per year) ALP based**			
Pre-SSE radium-223	6.04	5.23	6.91
Pre-SSE BSC	5.72	4.73	6.80
Post-SSE radium-223	9.99	7.74	12.52
Post-SSE BSC	11.09	5.80	18.06
**Progressed disease**			
Physician outpatient visit (per week)	0.25	0.20	0.30
Imaging CT scan – abdominal (per week)	0.08	0.06	0.10
Imaging bone scan (per week)	0.08	0.06	0.10
Complete blood count (per week)	0.08	0.06	0.10
Prostatic Specific Antigen (per week)	0.08	0.06	0.10
**Length of stay (days per year) ALP based**			
Pre-SSE radium-223	7.70	4.36	11.97
Pre-SSE BSC	12.94	7.51	19.83
Post-SSE radium-223	34.67	19.12	54.77
Post-SSE BSC	30.19	16.85	47.36
**Care Center Use (days per year) ALP based**			
Pre-SSE radium-223			
Pre-SSE BSC	0.02	0.00	0.07
Post-SSE radium-223	0.68	0.02	2.41
Post-SSE BSC	3.47	0.75	8.23
**Physician visits (visits per year) ALP based**			
Pre-SSE radium-223	9.68	5.67	14.75
Pre-SSE BSC	8.42	4.92	12.84
Post-SSE radium-223	13.97	5.58	26.14
Post-SSE BSC	9.84	6.46	13.92
**Unit costs**			
Radium-223	4532.50		
Hospitalization day cost	443.61	354.89	532.33
Day Care Center Cost per Day	186.26	149.01	223.51
Physician visits	81.02	64.82	97.22
End of life care	4181.67	3345.34	5018.00
External beam radiation	2427.72	1942.75	2913.26
**Outpatient costs for adverse events**			
Neutropenia	273.10	218.48	327.72
Febrile neutropenia	7168.10	5734.48	8601.72
Fatigue	0.00	0.00	0.00
Nausea	0.51	0.41	0.61
Vomiting	15.41	12.33	18.49
Anaemia	501.83	401.46	602.20
Thrombocytopenia	387.28	309.82	464.74
Hypokalemia	1.55	1.24	1.86
Bone Pain	453.60	362.88	544.32
Diarrhoea	12.00	9.60	14.40

ALP: alkaline phosphatase; BSC: best supportive care; CT: computed tomography; OWSA: one-way sensitivity analysis; SSE: symptomatic skeletal event

Note: Range tested in one-way sensitivity analysis was ± 20% over base case value

**Table 4:** Cost-effectiveness Analysis Base Case Results

	LYs	QALYs	Cost (€)	ICER(€/QALY)
BSC	1.48	0.77	55 437	
Radium-223	1.88	1.12	65 067	
Incremental	0.40	0.35	9631	27 606

BSC: best supportive care; ICER: incremental cost-effectiveness ratio; LY: life year; QALY: quality-adjusted life year

**Table 5:** Cost Breakdown Base-case Results

	Radium-223 (€)	Best Standard of Care (€)	Incremental (€)
**Cost breakdown**			
Drug costs	27 195	0	27 195
Cost of administration	348	0	348
Patient management costs	25 238	39 881	−14 643
Hospitalization cost	5027	8281	−3255
Day care center use cost	212	74	138
Physician visits	1082	977	106
Second & subsequent lines of treatment	654	1081	−427
End of life care	3958	4034	−76
SSE costs	1223	953	270
Drugs used AE costs	130	155	−25

AE: adverse event; SSE: symptomatic skeletal event

**Table 6:** Scenario Analysis Using Different Fitted Survival Curves

OS curve	ICER
Exponential	17 381 €/QALY
Log-logistic	25 242 €/QALY
Weibull	38 298 €/QALY
**PFS curve**	
Exponential	16 161 €/QALY
Log-logistic	46 989 €/QALY
Weibull	88 069 €/QALY

ICER: incremental cost-effectiveness ratio
